# Differences in the Response of Invasive *Solidago canadensis* and Native *Imperata cylindrica* to Glyphosate

**DOI:** 10.3390/plants14172640

**Published:** 2025-08-25

**Authors:** Xiaoqi Ye, Chunfeng Gu, Jinliu Meng, Ming Wu

**Affiliations:** 1 Research Institute of Subtropical Forestry, Chinese Academy of Forestry, Hangzhou 311400, China; mengxqi@caf.ac.cn (X.Y.); chunfeng.gu1@scbg.ac.cn (C.G.); mengjinliu0542@sina.com (J.M.); 2 National Research Station of Hangzhou Bay Wetlands Ecosystem, Hangzhou 311400, China; 3 State Key Laboratory of Wetland Conservation and Restoration, Chinese Academy of Forestry, Beijing 100091, China

**Keywords:** exotic invasive species, herbicide control, competition, *Solidago canadensis*, native species restoration

## Abstract

Exotic invasive plant species can cause biodiversity loss by outcompeting and replacing native species. Herbicides are commonly used to control invasive plants owing to their low cost and high efficiency. However, herbicide use can have unintended effects on co-occurring native plant species by altering the competitive balance. We studied how herbicide application modifies the competition between an invasive and a native species. We examined the effects of applying glyphosate on the mortality, photosynthetic capacity, and growth of *Solidago canadensis*, an aggressive invasive species, and *Imperata cylindrica*, a native species that commonly co-occurs with *S. canadensis*. We also studied how applying glyphosate affected the competition between these species. Various glyphosate concentrations were applied to the two species grown either together or separately. The mortality rate increased while the photosynthetic capacity and growth decreased with increasing glyphosate concentration. Increasing the glyphosate concentration more negatively affected the parameters of *I. cylindrica* than those of *S. canadensis*. Plant growth, especially that of *I. cylindrica,* was more restricted by intraspecific competition than by interspecific competition as the glyphosate concentration increased. Furthermore, the relative competitive potential of the native species decreased with increasing glyphosate concentration. *S. canadensis* is more tolerant of glyphosate, which enhances its competitive advantage and hinders the proliferation, reintroduction, and success of native plant species. Future studies should focus on developing techniques to mitigate the negative impacts of invasive plant species, for example, via optimizing methods of spraying herbicides.

## 1. Introduction

Global climate change has increased the invasiveness of exotic species in new areas and consequently leads to damages to local ecosystems [1,2,3,4,5]. In particular, many invasive plant species encroach on agricultural fields and natural reserves, where they directly compete with crops and native plants for space, nutrients, and pollinators during growth and reproduction, resulting in substantial yield and biodiversity losses [6,7]. Therefore, managing invasive species has become a major issue in agriculture and natural protection [8,9].

Various methods are used to control invasive species, with herbicides commonly used because of their efficiency and cost-effectiveness [10,11]. However, concerns have arisen about the secondary effects of the methods used to manage invasive species, particularly those related to herbicide use [12,13]. Herbicides can negatively affect the biodiversity of plants, microorganisms, fauna, and overall ecosystem functioning [14,15,16]. Herbicides reach and affect nontarget plants by drifting through the air [17], diffusing into the water of wetland ecosystems [18], or being released from the roots of target plants and transferring to nontarget plants [19,20]. The residues of some herbicides in the soil can have long-lasting effects depending on the timing of application [21]. Therefore, herbicides can have serious negative nontarget effects on native plant species.

The herbicides used to control invasive species can have negative, neutral, or positive effects on native species [22,23], depending on the type and dosage of the herbicide, as well as the context of the ecosystem being managed. A single herbicide application can cause the populations of native forbs to decline, thereby allowing for the establishment and spread of invasive species [14]. The abundance of native plants is negatively affected by 2,4-D ester or glyphosate application, whereas invasive monocots may thrive with their application [18,23]. In contrast, the native plant community and representative native species were not affected and only modestly affected by herbicides applied to control invasive species in other studies [22,24]. The responses of native plant communities and species to herbicides vary widely. Therefore, the mechanisms underlying the different responses must be understood. The short-term effects of herbicide application are mainly determined by the sensitivity and tolerance of native species to the herbicide [18,25,26]. Some invasive species are highly tolerant to herbicides [27,28]. For example, their photosynthetic capacity and growth may be less affected by herbicide application than those of native species [18,25]. This stronger herbicide tolerance may increase their competitiveness and hinder efforts to restore native plants to these habitats [14,29]. Therefore, the differences in the impacts of herbicides on the growth and competition of invasive and native species must be better understood.

Determining the mechanisms underlying the tolerance of individual species to herbicides provides a foundation for understanding the responses of entire plant communities to herbicides applied to eradicate invasive species. However, previous studies have not compared the herbicide tolerance between invasive and co-occurring native species or linked the differences in their interactions during regrowth periods. Therefore, this study focused on the simultaneous effects of herbicide application on invasive and native plant species to understand how attempts to restore native plant species are affected by herbicide application. Specifically, we aimed to determine the differences in the effects of herbicide application on mortality, photosynthetic capacity, growth, and competition. In this study, we aimed to clarify how herbicide application alters the competitive balance between invasive and native plants. We hypothesized that invasive species might be more tolerant to herbicides, thereby enabling a competitive advantage over native species.

*Solidago canadensis* (Canada goldenrod) is native to North America and has become invasive in Asia, Europe, and Australia [30,31,32]. *S. canadensis* rapidly expands through prominent clonal growth and the dispersal of large numbers of seeds. As an invasive species, *S. canadensis* frequently replaces native plant species through competition [33,34]. Herbicide application is the most common and cost-effective method of controlling *S. canadensis* [31,35]. However, the effects of the herbicides used to control *S. canadensis* in ecosystems remain unclear. Field tests have shown that glyphosate, a widely used herbicide [36,37], effectively controls this species but also harms various native species. Glyphosate application may alter the interactions between *S. canadensis* and native plant species during growth recovery periods, involving the reestablishment of native species. However, the effects of glyphosate have not been examined.

In this study, *Imperata cylindrica* (Poaceae) was selected as a representative native grass species because *I. cylindrica* is the dominant native grass species that coexists with *S. canadensis* in eastern China [38]. *I. cylindrica* is a fast-growing pioneer species that rapidly occupies available habitats and exhibits early resistance to invasion by *S. canadensis*. *I. cylindrica* is often replaced by *S. canadensis* in the field, and controlling *S. canadensis* with herbicides does not enable the successful re-establishment of *I. cylindrica*, suggesting the off-target effects of glyphosate. This study highlights the importance of considering the nontarget effects of herbicide application and provides a theoretical basis for determining whether herbicides can be applied to control exotic invasive species when reestablishing native species.

## 2. Results

### 2.1. Plant Mortality Rate in Glyphosate and Competition Treatments

The plant mortality rate significantly differed between species, at different glyphosate concentrations, and under competition (Table 1, *p* < 0.05). The mortality rate increased with increasing glyphosate concentration in both species (Table 1 and Supplementary Table S1) and was higher for *I. cylindrica* than for *S. canadensis*. *I. cylindrica* mortality was 66.0% higher at 1.8 mL·L^−1^ glyphosate when averaged for all the competition treatments. The mortality rate between species significantly differed in the 1.2–1.8 mL·L^−1^ glyphosate treatments (*p* < 0.05). *S. canadensis* showed a higher mortality rate in the mixture treatment than in the monoculture treatment (e.g., 38.6% higher at 1.8 mL·L^−1^ glyphosate), whereas *I. cylindrica* mortality showed a higher mortality rate in the monoculture treatment (e.g., 8.4% higher at 1.8 mL·L^−1^).

### 2.2. Response of Photosynthetic Capacity to Glyphosate Application and Competition

The glyphosate concentration and species competition affected the photosynthetic capacity of both species (Table 2) and more strongly affected the maximum net photosynthetic rate (P_nmax_) and light saturation point (LSP) of *I. cylindrica* (Supplementary Table S2). The stomatal conductance (G_s_) of *S. canadensis* was more affected by glyphosate and competition than that of *I. cylindrica*. The effects of the interaction between glyphosate and competition on P_nmax_ and G_s_ were significant for *S. canadensis* (*p* < 0.05).

**Figure 1:**
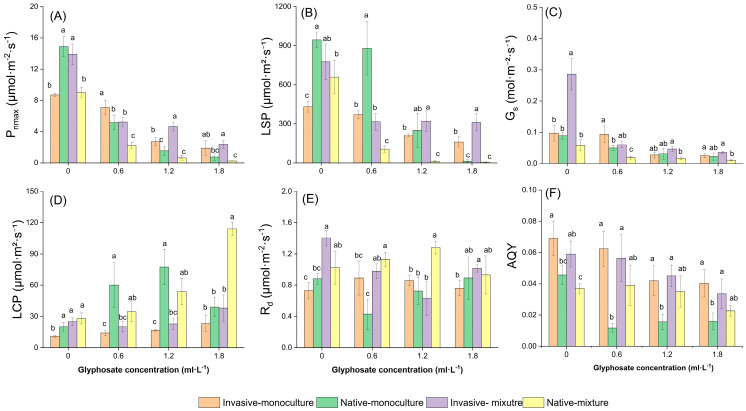
Effects of glyphosate application and competition on the photosynthetic parameters of the invasive *Solidago canadensis* and native *Imperata cylindrica*. Plants were grown either in monoculture (four *S. canadensis* plants or four *I. cylindrica* plants) or mixed (two *S. canadensis* plants + two *I. cylindrica* plants). (**A**) P_nmax_, maximum net photosynthetic rate; (**B**) LSP, light saturation point; (**C**) G_s_, stomatal conductance; (**D**) LCP, light compensation rate; (**E**) R_d_, dark respiration rate; (**F**) AQY, apparent quantum yield. Only significant differences between different combinations of species × competition for each parameter are shown for clarity. Different lowercase letters (a, b, and c) indicate significant differences (*p* < 0.05).

For both species, the P_nmax_, LSP, G_s,_ and apparent quantum efficiency (AQY) generally decreased, the light compensation point (LCP) increased, and the R_d_ remained relatively stable with increasing glyphosate concentrations (Figure 1). The P_nmax_ of *I. cylindrica* was higher than that of *S. canadensis* in the absence of glyphosate in the monoculture treatment. However, the P_nmax_ of *S. canadensis* was higher when glyphosate was applied and was consistently higher when the species were grown together (Figure 1A). The LSP of *I. cylindrica* was higher than that of *S. canadensis* in the monoculture treatment, except at a glyphosate concentration of 1.8 mL·L^−1^. However, the LSP of *S. canadensis* was higher when the species were grown together (Figure 1B). The G_s_ and AQY were generally higher and the LCP was lower for *S. canadensis* than for *I. cylindrica*, regardless of the competition treatment (Figure 1C,D). Changes in the R_d_ of the species were inconsistent among the glyphosate concentrations (Figure 1E).

Competition between species altered their photosynthetic capacities. The P_nmax_, LSP, and G_s_ of *S. canadensis* were higher under interspecific competition (mixed treatment) than under intraspecific competition (monoculture treatment), except for the 0.6 mL·L^−1^ glyphosate treatment. In contrast, the P_nmax_, LSP, and G_s_ of *I. cylindrica* were higher in the monoculture treatment than in the mixed treatment (Figure 1A–C). The LCP and R_d_ were generally higher under interspecific competition than under intraspecific competition for both species (Figure 1D,E). The AQY was lower for *S. canadensis* and higher for *I. cylindrica* under interspecific competition than under intraspecific competition (Figure 1F).

### 2.3. Plant Growth Response to Glyphosate and Competition

Both glyphosate and competition affected the growth and biomass accumulation of *S. canadensis* and *I. cylindrica* (Table 3). The total, aboveground, and belowground biomass of both species was significantly affected by glyphosate, competition type, and their interaction (*p* < 0.05). However, the number of green leaves of *S. canadensis* and the total green leaf length of *I. cylindrica* were significantly affected only by glyphosate treatment. Glyphosate and the interaction between glyphosate and interspecific competition generally more strongly affected *I. cylindrica* than *S. canadensis*, whereas the effects of interspecific competition were generally stronger in *S. canadensis*.

**Figure 2:**
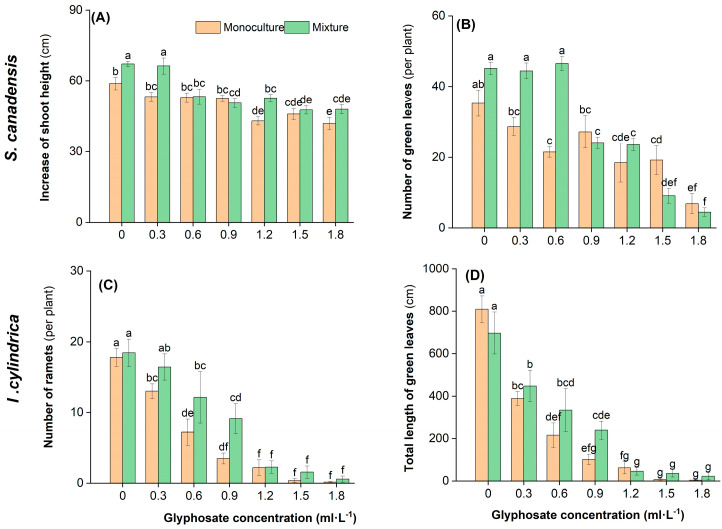
Effect of glyphosate application and competition on the growth of *Solidago canadensis* and *Imperata cylindrica*. Plants were grown either in monoculture (four *S. canadensis* plants or four *I. cylindrica* plants) or mixed (two *S. canadensis* plants + two *I. cylindrica* plants). (**A**) Increase in shoot height of *S. canadensis*; (**B**) number of green leaves of *S. canadensis*; (**C**) number of ramets of *I. cylindrica*; (**D**) total length of green leaves of *I. cylindrica*. Data are presented as the mean ± standard error (*n* = 7). Different letters indicate significant differences (*p* < 0.05) between treatments with different combinations of glyphosate concentration and competition.

**Figure 3:**
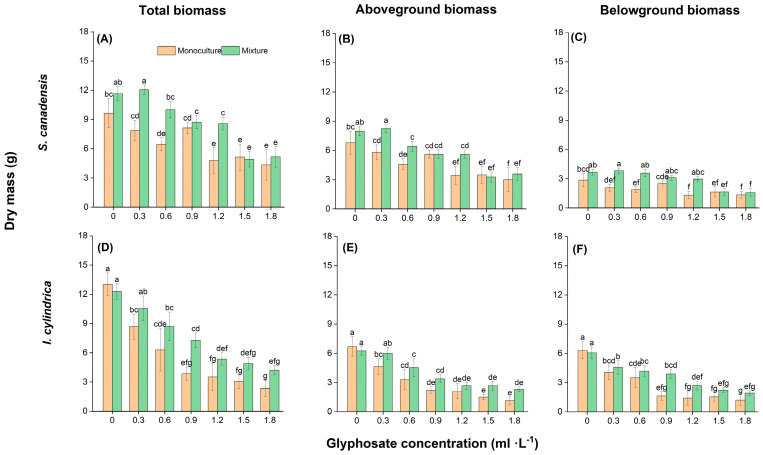
Effect of glyphosate application and competition on the biomass of *Solidago canadensis* and *Imperata cylindrica*. Plants were grown either in monoculture (four *S. canadensis* plants or four *I. cylindrica* plants) or mixed (two *S. canadensis* plants + two *I. cylindrica* plants). (**A**,**D**), (**B**,**E**), and (**C**,**F**) Total, aboveground, and belowground biomass of *S. canadensis and I. cylindrica*, respectively. Data are presented as mean ± standard error (*n* = 7). Different letters indicate significant differences (*p* < 0.05) between treatments with different combinations of glyphosate concentration and competition.

The growth of both species generally decreased with increasing glyphosate concentration (Figure 2 and Figure 3, Supplementary Table S1). This reduction was more pronounced in the monoculture treatment than in the mixed treatment and in *I. cylindrica* than in *S. canadensis* (Supplementary Table S2). The total plant biomass was lower by 33.3–81.8% and 18.5–54.9% in the monoculture treatment and by 14.1–65.6% and 3.4–55.7% than in the mixed treatment for *I. cylindrica* and *S. canadensis*, respectively. The reductions in the other growth parameters (e.g., aboveground biomass) with increasing glyphosate concentration were similar.

**Figure 4:**
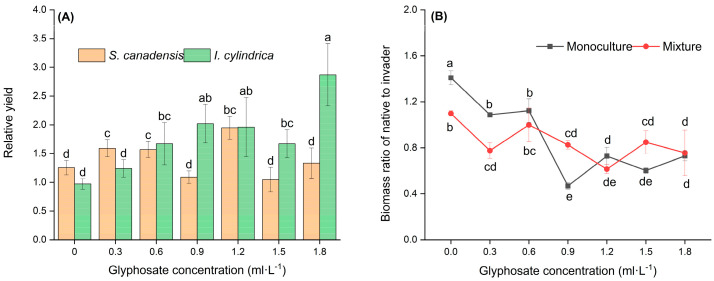
Effect of glyphosate application on competition between *Solidago canadensis* and *Imperata cylindrica*. The plants were grown in monoculture (four *S. canadensis* plants or four *I. cylindrica* plants) or mixed (two *S. canadensis* plants + two *I. cylindrica* plants). (**A**) Relative yield of *S. canadensis* and *I. cylindrica*; (**B**) Biomass ratio of *I. cylindrica* relative to that of *S. canadensis*, either in monoculture or mixed plots. Data are the mean ± standard error (*n* = 7). Different letters indicate significant differences (*p* < 0.05) between treatments with different combination of glyphosate concentration.

Interspecific competition generally led to increased growth in both species compared with that observed with intraspecific competition. The relative yield (RY) differed significantly between the species and glyphosate concentrations (F = 2.85, *p* < 0.05, Figure 4A). In both species, RY was higher at lower glyphosate concentrations and decreased as the glyphosate concentration increased. The RY of *I. cylindrica* was lower at 0–0.3 mL·L^−1^ and higher at 0.6–1.8 mL·L^−1^ than that of *S. canadensis*. The ratio of *I. cylindrica* to *S. canadensis* biomass decreased with increasing glyphosate concentrations under both intra- and interspecific competition (F = 11.828, *p* < 0.05; Figure 4B).

## 3. Discussion

The exotic invasive species *S. canadensis* was more tolerant to glyphosate than the native *I. cylindrica*. Applying glyphosate reduced the ability of *I. cylindrica* to compete with *S. canadensis*. Consequently, using glyphosate to control *S. canadensis* may suppress the populations of native *I. cylindrica* in the long term, particularly at sublethal doses. Therefore, strategies must be developed to mitigate these nontarget effects.

### 3.1. High Tolerance of S. canadensis to Glyphosate

The resistance of *S. canadensis* to glyphosate was much higher than that of *I. cylindrica,* as indicated by their photosynthetic and regrowth capacities. *S. canadensis* was not affected by moderate glyphosate application (below 0.6 mL·L^−1^) and was able to survive to a certain degree and regrow at a glyphosate concentration of 1.8 mL·L^−1^, indicating a high tolerance to glyphosate. Yanniccari et al. [39] reported that G_s_ is a sensitive indicator of glyphosate susceptibility in plants. However, our study showed that the P_nmax_ and LSP of *S. canadensis* and *I. cylindrica* were more sensitive than G_s_ to glyphosate application. The P_nmax_ and LSP of the invasive and native species showed different responses to glyphosate, and they were consistent with the trends in total biomass. The differences in the findings between studies could be due to differences in the duration between glyphosate treatment and photosynthesis measurement (three weeks in this study vs. 1–7 days in Yanniccari et al. [39]). Short-term photosynthesis changes may be more closely related to G_s_, whereas long-term plant growth may be limited by the number of photosynthetic apparatuses, as indicated by P_nmax_ and LSP [40]. Glyphosate inhibits the biosynthesis of tyrosine, phenylalanine, and tryptophan, which are three essential amino acids found in all proteins [36]. The combined influence of decreases in P_nmax_, LSP, G_S_, and AQY and increases in the LCP may reduce the carbon assimilation capacity and growth of plants. In this study, the growth of the above- and belowground parts of the two species was similarly affected by glyphosate, which is transported from the leaves to the roots and rhizomes [36]. However, the systemic responses of plants to herbicides may not be caused by direct injury to each part but rather by resource allocation to the aboveground and belowground parts.

This study only evaluated the tolerance of one species; however, herbicide resistance varies widely among and within species [21,25,41]. *S. canadensis* might be more tolerant to herbicides than most co-occurring native species because many invasive species are more stress-tolerant and more quickly regrow after disturbances [42,43,44]. High herbicide tolerance is frequently observed in weeds and exotic plant species [27,28], which is likely due to the selection of resistant genotypes through herbicide application in these species [45]. Invasive plant species rapidly expand from their native range to an invasive range [46,47]. The high herbicide tolerance in some invasive species is due to mutations [45], as recorded in many weeds [41]. Polyploidization may also enhance tolerance to herbicides [30,48]. The mechanisms of herbicide tolerance differ within invasive species and may not be mutually exclusive. First, exotic invasive species may have a stronger capacity to biotransform, degrade, or detoxify herbicides, thereby reducing the associated damage [49,50]. Second, exotic invasive plant species tend to be larger than the native species they replace [51,52] and thus may require higher amounts of herbicides to cause equivalent levels of damage. Third, if both species have similar clonal growth forms, then the invasive plant species may benefit more from clonal integration than native plant species under various stress conditions [53,54]. In this study, the higher clonal integration of *S. canadensis* compared with that of *I. cylindrica* provided more protection against herbicide damage; however, in a previous study on the invasive plant *Carpobrotus eduli,* the level of clonal integration did not enhance the plant’s performance after glyphosate treatment [55]. Finally, invasive plant species that show stronger associations with soil microorganisms (such as arbuscular mycorrhizal fungi) than native species may be more tolerant to herbicides [56].

### 3.2. Glyphosate Treatment Altered Competition Between S. canadensis and I. cylindrica

Although native vegetation attempts to resist invasions by exotic plant species [57,58], invasive species outcompete native species for resources and have stronger reproductive capacities [51,59], resulting in the replacement of native species with invasive species [60,61]. We found that the application of herbicides to control invasive species may alter the interactions between invasive and native species, influencing attempts to re-establish native species. The higher RY of *S. canadensis* indicated that this species benefited more from reductions in intraspecific competition than the native species when herbicide was not applied. This partially explains the higher invasive capacity of native grasses [38,62]. Conversely, the higher RY of *S. canadensis* after glyphosate application indicates that the native *I. cylindrica* is less negatively affected when competing with *S. canadensis* due to reduced intraspecific competition intensity. A model study also indicated that native plant species experience a relief period after herbicide degradation and before invasive species re-establishment [29]. However, the competitive ability of *I. cylindrica* decreased with increasing glyphosate concentrations during the regrowth period, as indicated by the decrease in the ratio of *I. cylindrica* to *S. canadensis* biomass. This reduction in the competitive ability of *I. cylindrica* was attributed to its lower tolerance to glyphosate. These findings suggest that the application of herbicides to invasive species during the growing season further decreases the population of *I. cylindrica* at sites with *S. canadensis*. In addition to the differences in tolerance between these nontarget native and target invasive species, herbicide application may encourage secondary invasion. The failure of native vegetation to re-establish following herbicide application may occur because of re-invasion by the target invasive species or the establishment of other non-native plants [23,63]. Many invasive species can colonize disturbed sites more rapidly than native species because they have a stronger ability to regrow after disturbances [64] and greater number of propagules available for colonization [65,66].

This study was limited to two months, and only one dominant species was considered. Whether the differences in the effects of herbicide application persist for longer durations under field conditions remains unclear. Differences in herbicide tolerance and competitive capacity between invasive species can alter the response of the entire plant community. Long-term evaluations are important for quantifying the outcomes of attempts to restore native species [11,29]. Glyphosate application may temporarily alleviate competition intensity by suppressing the growth of both invasive and native species [67]; however, competition intensity may strengthen as invasive species recover from herbicide stresses, as predicted by the stress gradient hypothesis [68,69]. This may explain why some native plant species show neutral or positive dominance to herbicide application in the short term [22,23,24] but then exhibit decreased dominance in the long term [14,16]. Further long-term field studies of the community-level interactions between native and invasive species following herbicide application are essential for guiding the successful restoration of native vegetation.

### 3.3. Potential Approaches for Alleviating Nontarget Effects of Herbicides

Several approaches can be used for reducing the nontarget effects of herbicides on native plant species: (1) Spray methods can be optimized. Applying herbicides using spot or boom sprays based on the density of the target invasive species may reduce injury to native species [70]. (2) The application of control methods can be more precisely timed. Native and invasive plant species show distinct phenological niche separation in some cases, which allows for the control of invasive species without harming native species through the precise timing of herbicide application. For example, the winter application of herbicides effectively suppressed the regrowth of winter-active *S. canadensis* but did not impact the growth of winter-dormant *I. cylindrica* in a subtropical climate [71]. (3) The propagules of native plant species can be protected. Providing sufficient propagules [35] and protecting seeds via pelleting with local soil or activated carbon after herbicide application can enhance the restoration ability of native species [72,73]. However, the efficiencies of these approaches are highly dependent on the context; therefore, further study is required.

## 4. Materials and Methods

### 4.1. Seedling Culture

The seedlings were collected from a nature conservation area in the Hangzhou Bay National Wetland Park (121°08′43″ E, 30°18′40″ N) in Ningbo, Zhejiang Province, China. Individual ramets with five mature leaves and a shoot height of 13–15 cm were chosen for *S. canadensis* and *I. cylindrica* on 20 March 2016. The rhizomes were uniformly trimmed to 2–3 cm in length. The plants were temporarily grown in river sand and watered with tap water for three weeks. Plants of similar sizes and growth vigor were transplanted into pots (23 × 17 cm). The pots were filled with soil collected from the same site where the seedlings were collected. The soil was silty, characterized by mild salinity (2.5‰), slightly alkaline conditions (pH 8.2), low fertility (0.45 g·kg^−1^ total N, 0.60 g·kg^−1^ total P, and 7.23 g·kg^−1^ total soil organic matter content), and a field water holding capacity of 28.9%. We added 2 g of urea and 2 g of potassium dihydrogen phosphate (KH_2_PO_4_) to each pot to mitigate nitrogen and phosphorus limitations from affecting plant growth. The plants were acclimatized to greenhouse conditions for three weeks prior to the start of the treatments.

### 4.2. Competition and Glyphosate Treatments

A two-factor experiment involving competition and glyphosate application was conducted in a greenhouse at the Institute of Subtropical Forestry of the Chinese Academy of Forestry. A replacement design [74] was used for the competition treatments: four *S. canadensis* plants (*S. canadensis* monoculture) or four *I. cylindrica* plants (*I. cylindrica* monoculture) were grown without interspecific competition within a plastic pot or two *S. canadensis* plants and two *I. cylindrica* plants were grown together (mixed treatment, with interspecific competition). The effects of applying different glyphosate concentrations on *S. canadensis* leaf chlorosis and withering were investigated. Applying 1.8 mL·L^−1^ glyphosate caused serious leaf injury and plant death. Each competition treatment included one of seven glyphosate concentration levels: 0, 0.3, 0.6, 0.9, 1.2, 1.5, or 1.8 mL·L^−1^ (41% Roundup [Monsanto Company, Saint Louis, MO, USA] in distilled water), with 5 mL of glyphosate solution applied per pot to examine the drift effects of sublethal glyphosate dosages on the two species. The glyphosate dosages were much lower than those applied to achieve effective control in the field [75] owing to the large differences in plant size between the species investigated in this study and those growing in field. Glyphosate solutions were evenly sprayed on the leaves of each plant using a 20 mL plastic sprayer on 12 June 2016. Seven replicates were conducted for each combination of competition and glyphosate treatment. A total of 147 pots were used (seven glyphosate concentration treatments × three competition treatments × seven replicates). The pots were rearranged once per week to minimize the effects of environmental heterogeneity within the greenhouse. The photosynthetically active radiation (PAR) intensity in the greenhouse was approximately 50–70% of the ambient PAR depending on the external PAR. The air temperature and relative humidity in the greenhouse followed external conditions. The temperature fluctuated between 20 and 38 °C during the day and between 20 and 26 °C at night, and the relative humidity was 40–95%. The plants were watered regularly to avoid drought stress.

### 4.3. Photosynthesis Measurements

The growth of the treated plants was monitored starting on the first day of glyphosate treatment. Symptoms of injury and regrowth were observed 10 and 20 days after glyphosate spraying, respectively. Photosynthetic capacity was a major determinant of regrowth, and it was measured using a 6400 XT Portable Photosynthesis Measurement System (LiCOR, Lincoln, NE, USA) after three weeks of treatment. Before this, the first three fully expanded *S. canadensis* and *I. cylindrica* leaves of the same position were selected from each plant. Three individual plants of *S. canadensis* and *I. cylindrica* from either the monoculture or mixed treatment at each glyphosate concentration were selected for testing on sunny days between 7:30 and 11:30. The photosynthetic parameters were measured along a PAR gradient of 1500, 1200, 1000, 800, 600, 400, 200, 150, 100, 50, 20, and 0 μmol·m^−2^·s^−1^. The PAR–P_n_ curves were fitted using a nonrectangular hyperbolic equation to determine the P_nmax_, LSP, LCP, AQY, and dark respiration rate (R_d_) [76]. The Gs was recorded using the PAR at the LSP. The values of the photosynthetic parameters obtained from three leaves of the same plant were averaged before statistical analysis.

### 4.4. Measurement of Mortality, Growth, and Competition Effects

The growth and mortality rates were evaluated after two months of growth. Shoot height and number of green leaves were recorded for each *S. canadensis* plant, whereas the number of ramets and total length of green leaves were recorded for *I. cylindrica*. The mortality rate was calculated in each pot by assessing the presence of green leaves, and it was defined as the ratio of dead plants to the total number of plants (four in the monoculture treatment and two in the mixed treatment). Biomass of surviving live plants in at least three pots was analyzed. Mortality rates were averaged for each replicate of each combination treatment. Plants were harvested to determine the biomass on August 18–20, 2016. The belowground parts of each plant were washed and separated from the aboveground parts. All the harvested plants were dried at 80 °C to a constant weight. The RY was used to assess the relative intensity of interspecific competition with intraspecific competition based on total individual biomass [74,77]. The RYs of *S. canadensis* and *I. cylindrica* were calculated using the following formula:

RY = Y_mix_/Y_mono_

where Y_mix_ indicates the total *S. canadensis* or *I. cylindrica* biomass in each pot in the mixed treatment and Y_mono_ indicates the total biomass of the corresponding species in each pot in the monoculture treatment.

The ratio of *I. cylindrica* to *S. canadensis* biomass was calculated to evaluate the relative competitive capacities of the species. The *I. cylindrica* and *S. canadensis* plants grown in the same pot were paired in the mixed treatment. The *I. cylindrica* and *S. canadensis* plants from the same glyphosate treatment group were randomly paired in the monoculture treatment.

### 4.5. Data Analysis and Statistics

The normality of all data was tested using the Shapiro–Wilk test. Two-way analysis of variance (ANOVA) was conducted to analyze the general effects of glyphosate concentration, competition, and their interactions on photosynthetic parameters (P_nmax_, LSP, Gs at LSP, LCP AQY, and R_d_), growth (increase in shoot height and number of green leaves of *S. canadensis*; number of ramets and total length of green leaves of *I. cylindrica*), and biomass (aboveground, belowground, and total biomass of *S. canadensis* and *I. cylindrica*). One-way ANOVA was performed for the above parameters to determine the differences between the combined glyphosate × competition treatments. The data were transformed using the square root, natural log, or arcsine when the assumption of homogeneity of variance was not met. One-way ANOVA was also used to test the effects of glyphosate concentration on the mortality rate and the ratio of *I. cylindrica* to *S. canadensis* biomass for each glyphosate concentration treatment in the mixed or monoculture treatment. Multiple comparisons were performed using the Duncan method to determine the significance of the differences between the different treatment combinations. The size of these effects was estimated using the partial eta squared (Supplementary Table S2). A regression analysis between the glyphosate concentration and mortality rate as well as the growth parameters (growth and biomass) was also performed to examine the trends in these parameters with increasing glyphosate concentration (Supplementary Table S1). Statistical analyses were conducted using IBM SPSS Statistics 27 (Armonk, NY, USA).

## 5. Conclusions

Glyphosate applied for the control of the invasive species *S. canadensis* has nontarget effects on native plant species. The lower tolerance of *I. cylindrica* to glyphosate was evidenced by its higher mortality rates, decreased photosynthetic capacity, and suppressed regrowth capacity compared with those of *S. canadensis*. Moreover, the lower tolerance decreased the relative competitive ability of *I. cylindrica* with increasing glyphosate dosage. This could ultimately result in a decline in the population of this native species, thereby contradicting the intended goal of restoring native species by controlling invasive species. These nontarget effects may become more pronounced over time. Therefore, long-term studies on the responses of entire plant communities to herbicide application are essential for ensuring the effective management of exotic invasive species. Strategies must be developed and implemented to mitigate these adverse effects and support efforts to re-establish native plant species.

## Figures and Tables

**Table 1:** Plant mortality rate (%) of *Solidago canadensis* or *Imperata cylindrica* treated with different concentrations of glyphosate.

Glyphosate Treatments (ml·L^−1^)	*S. canadensis* Monoculture	*S. canadensis* Mixture	*I. cylindrica* Monoculture	*I. cylindrica* Mixture
0	0.0 ± 0.0 bA	0.0 ± 0.0 bA	0.0 ± 0.0 cA	0.0 ± 0.0 cA
0.3	0.0 ± 0.0 bA	0.0 ± 0.0 bA	0.0 ± 0.0 cA	0.0 ± 0.0 cA
0.6	14.3 ± 10.7 bA	0.0 ± 0.0 bB	14.3 ± 9.2 bA	14.3 ± 9.2 cA
0.9	7.1 ± 4.6 bB	7.1 ± 2.1 bB	39.3 ± 12.0 bA	7.1 ± 7.1 cB
1.2	25.0 ± 14.4 abB	0.0 ± 0.0 bC	71.4 ± 12.7 aA	50.0 ± 15.4 bA
1.5	21.4 ± 8.5 abC	50.0 ± 10.9 aB	89.3 ± 7.4 aA	57.1 ± 14.9 abB
1.8	46.4 ± 14.9 aB	64.3 ± 14.3 aB	92.9 ± 7.1 aA	85.7 ± 9.2 aA

Different lowercase letters (a, b, and c) indicate significant differences (*p* < 0.05) among glyphosate concentration treatments within each species × competition combination, whereas different uppercase letters (A, B, and C) indicate significant differences among different species × competition combinations within each glyphosate concentration treatment.

**Table 2:** Effects of competition and glyphosate treatment on photosynthetic parameters of *Solidago canadensis* and *Imperata cylindrica*.

Treatments	Species		Photosynthetic Parameters					
			P_nmax_	LSP	G_S_	LCP	R_d_	AQY
Glyphosate (G)	*S. canadensis*	*F*	31.1	11.4	20.985	2.1	2.1	1.0
		*P*	0.000 **	0.000 **	0.000 **	0.047 *	0.146	0.423
	*I. cylindrica*	*F*	44.1	18.9	10.399	2.2	1.5	3.3
		*P*	0.000 **	0.000 **	0.000 **	0.026 *	0.25	0.046 *
Competition (C)	*S. canadensis*	*F*	7.9	8.8	8.05	3.5	4.5	1.7
		*P*	0.013 *	0.009 *	0.012 *	0.081	0.05	0.213
	*I. cylindrica*	*F*	10.9	17.8	7.8	0.03	19.8	11.5
		*P*	0.005 **	0.001 **	0.013 *	0.874	0.009 **	0.004 **
G × C	*S. canadensis*	*F*	6.1	2.5	8.986	0.64	4.1	0.8
		*P*	0.006 **	0.094	0.001 **	0.602	0.065	0.513
	*I. cylindrica*	*F*	2.3	2.9	0.417	2.6	0.5	1.7
		*P*	0.112	0.066	0.743	0.086	0.656	0.204

Asterisks indicate significant differences in the treatments (G, glyphosate; C, competition; and G × C, interaction between glyphosate and competition) for each photosynthetic parameter. *, *p* < 0.05, **, *p* < 0.01. P_nmax_, maximum net photosynthetic rate; LSP, light saturation point; G_s_, stomatal conductance; LCP, light compensation rate; R_d_, dark respiration rate; AQY, apparent quantum yield.

**Table 3:** Effects of glyphosate treatment and competition on the growth and biomass accumulation of *Solidago canadensis* and *Imperata cylindrica*.

*S. canadensis*						
Treatments	Significance	Total biomass	Aboveground biomass	Belowground biomass	Shoot height	Number of green leaves
Glyphosate (G)	*F*	20.4	20.2	11.8	18.0	11.2
	*P*	<0.001	<0.001	<0.001	<0.001	<0.001
Competition (C)	*F*	69.7	82.6	22.4	18.5	0.6
	*P*	<0.001	<0.001	<0.001	<0.001	0.439
G × C	*F*	2.9	0.5	2.9	3.0	1.6
	*P*	0.011	0.03	0.013	0.01	0.151
***I. cylindrica***						
Treatments	Significance	Total biomass	Aboveground biomass	Belowground biomass	Number of ramets	Total length of green leaves
Glyphosate (G)	*F*	37.7	29.1	36.9	50.8	52.523
	*P*	<0.001	<0.001	<0.001	<0.001	<0.001
Competition (C)	*F*	37.8	32.2	36.9	13.2	1.379
	*P*	<0.001	<0.001	<0.001	<0.001	0.244
G × C	*F*	7.8	5.6	8.7	5.8	1.254
	*P*	<0.001	<0.001	<0.001	<0.001	0.288

## Data Availability

The data are available upon request.

## Supplementary Materials

The following supporting information can be downloaded at: https://www.mdpi.com/article/10.3390/plants14172640/s1, Table S1: Linear regression of the growth parameters with the glyphosate concentration for *S. canadensis* and *Imperata cylindrica*, Table S2: Effect size of the effects of the competition treatments and the glyphosate treatments on photosynthetic and growth parameters of *Solidago canadensis* and *Imperata cylindrica*.
