# Supplementary material for: Differences in the Response of Invasive Solidago canadensis and Native Imperata cylindrica to Glyphosate
Source: Plants (Basel). 2025 Aug 25;14(17):2640. doi: 10.3390/plants14172640 (PMC12430325; doi:10.3390/plants14172640)
Supplement: Supplementary file 1 [file plants-14-02640-s001.zip › Supplementary Table S1. .pdf]

**Supplementary Table S1.** Linear regression of the growth parameters with the glyphosate concentration for *S. canadensis* and *Imperata cylindrica.*

| | ***S. canadensis*** | | ***I. cylindrica*** | |
|---|---|---|---|---|
| | **Monoculture** | **Mixture** | **Monoculture** | **Mixture** |
| Mortality rate | $y = 22.9 \times x - 4.3$, $R^2 = 0.815$, $p = 0.005$ | $y = 34.9 \times x - 14.0$, $R^2 = 0.669$, $p = 0.025$ | $y = 61.2 \times x - 11.2$, $R^2 = 0.948$, $p < 0.001$ | $y = 48.5 \times x - 13.0$, $R^2 = 0.869$, $p = 0.002$ |
| Shoot height | $y = -8.9 \times x + 57.8$, $R^2 = 0.864$, $p = 0.002$ | $y = -11.3 \times x + 65.3$, $R^2 = 0.800$, $p = 0.007$ | - | - |
| Number of green leaves | $y = -12.8 \times x + 33.9$, $R^2 = 0.828$, $p = 0.004$ | $y = -25.6 \times x + 51.2$, $R^2 = 0.896$, $p = 0.001$ | - | - |
| Total length of green leaves | - | - | $y = -396.8 \times x + 584.3$, $R^2 = 0.784$, $p = 0.008$ | $y = -373.5 \times x + 596.6$, $R^2 = 0.913$, $p < 0.001$ |
| Number of ramets | - | - | $y = -9.9 \times x + 15.2$, $R^2 = 0.898$, $p = 0.001$ | $y = -11.1 \times x + 18.6$, $R^2 = 0.955$, $p < 0.001$ |
| Aboveground biomass | $y = -2.0 \times x + 6.5$, $R^2 = 0.847$, $p = 0.003$ | $y = -2.8 \times x + 8.4$, $R^2 = 0.91$, $p < 0.001$ | $y = -2.9 \times x + 5.7$, $R^2 = 0.886$, $p = 0.002$ | $y = -2.4 \times x + 6.1$, $R^2 = 0.917$, $p < 0.001$ |
| Belowground biomass | $y = -0.7 \times x + 2.6$, $R^2 = 0.626$, $p = 0.034$ | $y = -1.3 \times x + 4.1$, $R^2 = 0.858$, $p = 0.003$ | $y = -2.7 \times x + 5.2$, $R^2 = 0.821$, $p = 0.005$ | $y = -2.2 \times x + 5.6$, $R^2 = 0.955$, $p = <0.001$ |
| Total biomass | $y = -2.7 \times x + 9.1$, $R^2 = 0.797$, $p = 0.007$ | $y = -4.2 \times x + 12.5$, $R^2 = 0.911$, $p < 0.001$ | $y = -5.5 \times x + 10.8$, $R^2 = 0.849$, $p = 0.003$ | $y = -4.6 \times x + 11.8$, $R^2 = 0.967$, $p < 0.001$ |

Note: Y stands for growth parameter and x stands for glyphosate concentration (0, 0.3, 0.6, 0.9, 1.2, 1.5, and 1.8 ml·$L^{-1}$). The plants were either grown in monoculture (Four *S. canadensis* plants or four *I. cylindrica* plants) or in mixture (Two *S. canadensis* plants + two *I. cylindrica* plants).
