# Supplementary material for: Differences in the Response of Invasive Solidago canadensis and Native Imperata cylindrica to Glyphosate
Source: Plants (Basel). 2025 Aug 25;14(17):2640. doi: 10.3390/plants14172640 (PMC12430325; doi:10.3390/plants14172640)
Supplement: Supplementary file 1 [file plants-14-02640-s001.zip › Supplementary Table S2. .pdf]

Supplementary Table S2. Effect size of the effects of the competition treatments and the glyphosate treatments on photosynthetic and growth parameters of *Solidago canadensis* and *Imperata cylindrica.*

	**Glyphosate**	**Competition**	**Competition × Glyphosate**
	Solidago canadensis		
P_{nmax}	0.885	0.33	0.534
LSP	0.681	0.355	0.322
G_s	0.797	0.335	0.628
LCP	0.285	0.178	0.107
AQY	0.156	0.095	0.13
R_d	0.279	0.219	0.433
Shoot height	0.139	0.532	0.17
Number of green leaves	0.055	0.592	0.255
Total biomass	0.277	0.61	0.189
Aboveground biomass	0.192	0.622	0.149
Belowground biomass	0.327	0.46	0.197
	Imperata cylindrica		
P_{nmax}	0.891	0.393	0.31
LSP	0.78	0.526	0.353
G_s	0.661	0.328	0.072
LCP	0.293	0.002	0.33
AQY	0.386	0.418	0.243
R_d	0.093	0.326	0.221
Number of ramets	0.738	0.084	0.071
Total leaf length	0.792	0.017	0.085
Total biomass	0.703	0.159	0.074
Aboveground biomass	0.656	0.12	0.057
Belowground biomass	0.656	0.144	0.098

Note: The effect size were assessed by Partial eta-squared. For each species, the plants were exposed to two-factor treatments: competition and glyphosate. There were two competition treatments: with intraspecific competition (Four *S. canadensis* plants or four *I. cylindrica* plants) or with interspecific competition (Two *S. canadensis* plants + two *I. cylindrica* plants). For each competition treatment, the plants were treated with seven glyphosate concentration (0, 0.3, 0.6, 0.9, 1.2, 1.5, and 1.8 $ml \cdot L^{-1}$).
